# Preliminary results using a kit to measure tamoxifen and metabolites concentrations in capillary blood samples from women with breast cancer

**DOI:** 10.1038/s41598-022-05443-0

**Published:** 2022-01-31

**Authors:** Stefan Rehnmark, Ivan Shabo, Håkan Randahl, Yvonne Wengström, Per Rydberg, Elham Hedayati

**Affiliations:** 1 Redhot Diagnostics AB, Stockholm, Sweden; 2 Patient Area of Breast Cancer, Sarcoma and Endocrine Tumors, Theme Cancer, Karolinska University Hospital, Stockholm, Sweden; 3 Karolinska Comprehensive Cancer Center, Theme Cancer, Karolinska University Hospital, Stockholm, Sweden; 4 Division of Nursing, Department of Neurobiology, Care Sciences and Society (NVS), Karolinska Institute, Stockholm, Sweden; 5 Department of Oncology-Pathology, Karolinska Institute, Stockholm, Sweden; 6 Department of Oncology-Pathology, Cancer Center Karolinska, Karolinska Institutet, K7 Onkologi-Patologi, K7 OnkPat Bergh, 171 77 Stockholm, Sweden

**Keywords:** Biotechnology, Oncology

## Abstract

The aim of the study was to compare 3 blood sampling methods, including capillary blood sampling, for determining Tamoxifen (TAM), Z-endoxifen (END), and 4-hydroxytamoxifen (4HT) concentrations. High performance liquid chromatography-mass spectrometry was used to quantify concentrations of TAM, END, and 4HT in plasma, venous blood, and capillary blood samples of 16 participants on TAM therapy for breast cancer. The rhelise kit was used for capillary sampling. Calibration curves using ^13^C-labeled analogs of TAM, END, and 4HT as internal standards were used for quantifications. A capillary sampling kit was used successfully for all participants. Mean TAM concentrations did not differ significantly in the 3 types of samples. Mean END and 4HT concentrations did differ significantly between capillary and venous blood samples, possibly related to photodegradation in the internal standards prior to use or degradation products with chromatographic retention times similar to the metabolites. TAM, END, and 4HT concentrations were relatively stable when stored for 14 days at 8 °C and 20 °C. Therapeutic drug monitoring of TAM using an innovative kit and capillary blood sampling is feasible. Preliminary data from this study will aid in developing a multicenter, randomized clinical trial of personalized TAM dose monitoring and adjustments, with the goal of enhancing the quality-of-life and outcomes of patients with breast cancer.

**Clinical Trial Identification:** EudraCT No 2017-000641-44.

## Introduction

Tamoxifen (TAM) is a selective estrogen receptor modulator (SERM) that inhibits estrogen receptor (ER) transcriptional activity by binding to estrogen receptors. It is commonly used as adjuvant monotherapy to treat hormone-receptor (HR)-positive breast cancer (BC) in premenopausal women and as part of sequential therapy to treat BC in postmenopausal women.

BC is the most common cancer among Swedish women. At the present time, TAM is being prescribed to about 17,000 Swedish women, the majority of whom have received it as adjuvant therapy for BC^1^. The use of TAM 20 mg daily for 10 years has been shown to reduce BC recurrence rates by half and BC mortality rates by a third^2,3^. However, despite the well-known protective benefits of TAM therapy for women with BC, it is estimated that 50% of women discontinue the medication within the first few years, primarily because of the severity of adverse effects^4–6^. The high rate of discontinuation is a major drawback to the use of TAM therapy.

Currently, there are no routine therapeutic monitoring tests used for TAM or its active metabolites. Such tests, particularly if easily administered, could be useful in addressing the discontinuation, noncompliance, and adverse effect issues involving TAM therapy. Accurate and easily obtained quantification of concentrations of TAM and its metabolites could aid in identifying potentially modifiable factors contributing to adverse effects, including liver function abnormalities, polymorphisms in pertinent cytochrome P450 (CYP450) enzymes, and drug-drug interactions.

Therapeutic drug monitoring (TDM) is the process of analyzing drug concentrations in plasma or blood. TDM may also be defined as the clinical laboratory measurement of a chemical parameter that, with appropriate medical interpretation, can be used for the individualization of drug dosing. Using TDM, the prescribed dose of a drug can be adjusted within a targeted therapeutic range or window, titrating levels for both efficacy and side effects. TDM is particularly helpful for patients receiving drugs that have a narrow therapeutic window, for whom there are substantial risks of under- or overdosing. It is also useful for patients receiving drugs that must be bioconverted to be active and that have high inter-individual variability in their conversion rates.

In TAM metabolism, a series of CYP450 enzymes convert TAM to its two most active metabolites, Z-endoxifen (END) and 4-hydroxytamoxifen (4HT), and this conversion process may vary between patients^7^. The use of TDM for TAM and its active metabolites during TAM therapy has substantial theoretical appeal, particularly in light of the adverse effect, noncompliance, and discontinuation issues discussed above, but to date it has not been employed in routine clinical practice.

The preferred method for TDM has involved the use of Liquid Chromatography Mass Spectrometry (LC–MS/MS), which has a very high specificity and sensitivity and can be performed using small samples^8^. When TAM concentrations have been measured, the gold standard has been to use patient plasma samples with LC–MS/MS. However, plasma is less stable and has greater variability in the critical components that bind pharmaceutical drugs (e.g., albumin and lipids) than whole blood^9^. Thus, whether obtained with venipuncture or with capillary sampling methods, whole blood should be more suitable for TAM measurements than plasma. In actual clinical practice, conventional venous blood sampling has been used most often for TDM. However, this approach is associated with higher costs for healthcare materials and staff, more patient time and lost wages, and transportation challenges. On the other hand, capillary blood sampling may be preferable, as it is low-cost, easy-to-use, and can be done anywhere.

We hypothesized that TDM during TAM therapy may be feasible and that capillary blood sampling could be a viable alternative to venipuncture for performing TDM while patients are on TAM therapy. As a result, we participated in the development of a capillary blood sampling kit, the rhelise kit (Redhot Diagnostics, Sweden)^10^, to be used for TDM in patients on TAM therapy. This kit consists of a lancet, capillary tube (20 μL to 70 μL), and specimen vial containing a pre-mixed extraction solution.

In this study, we were interested in investigating the feasibility of using capillary blood sampling for TDM in patients with BC on TAM therapy. To do this, our first aim was to determine whether the concentrations of TAM and two of its active metabolites, END and 4HT, were similar in plasma and venous blood. Our next aim was to determine whether the concentrations of these substances were also similar in venous blood and capillary blood. In addition, we aimed to determine whether it was feasible to use the rhelise kit to obtain the capillary blood samples. Finally, we aimed to determine whether it was also feasible to use the extraction solution provided in the kit for measurements of TAM and its metabolites, which we did by measuring the stability over time and at different temperatures of blood samples after being mixed with the extraction solution.

## Results

### Patient characteristics

The study included 16 female participants whose median age was 50.0 (range, 35.0 to 82.0) years. The median duration of participants taking TAM was 18.5 (interquartile range [IQR], 11.0 to 22.0) months. Plasma, venous blood, and capillary blood samples were collected from all 16 participants at the same time by a trained research nurse. A total of 13 of the participants reported on their functioning and symptoms (Table 1).

**Table 1:** Participant-reported functioning and symptom measures in 13 participants with breast cancer who were surveyed at the time of sample submission.

	Questionnaire scores, median (IQR)
**EORTC core quality of life questionnaire (QLQ C-30)**	
Functioning	
Overall wellbeing	75 (67–83)
Physical functioning	93 (93–100)
Role functioning	100 (83–100)
Ability to work	1 (1–2)
Emotional functioning	67 (67–92)
Cognitive functioning	83 (83–100)
Social functioning	83 (67–100)
Financial impact	0 (0–0)
Symptoms	
Worry	2 (1–3)
Depressed	2 (1–2)
Pain	17 (0–33)
Fatigue	33 (22–33)
Insomnia	33 (33–67)
Nausea and vomiting	0 (0–0)
Dyspnea	33 (0–33)
Appetite loss	0 (0–0)
Constipation	0 (0–33)
Diarrhea	0 (0–67)
**EORTC breast cancer-specific quality of life questionnaire (QLQ-BR23)**	
Functioning	
Sexual functioning	17 (0–33)
Body image	67 (33–67)
Symptoms	
Arm symptoms	11 (0–22)
Hot flushes	4 (3–4)
**EORTC quality of life questionnaire liver module (QLQ-LMC21)**	
Symptoms	
Peripheral neuropathy	33 (33–67)
**Functional assessment of cancer therapy endocrine subscale (FACT-ES)**	
Symptoms	
Vaginal symptoms	50 (38–57)
Arthralgia	2 (1–2)
**Measures of satisfaction with breast**	
BREAST-Q mastectomy	53 (48.5–62)
BREAST-Q breast conserving Therapy	68 (59.5–97)

EORTC, European Organization for Research and Treatment of Cancer; IQR, interquartile range.

### Plasma vs. venous blood concentrations of TAM, END, and 4HT

**Figure 1:**
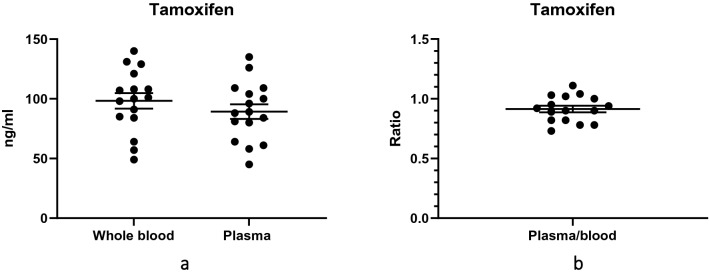
Tamoxifen (TAM) concentrations in 16 participants with breast cancer taking tamoxifen 20 mg daily: (**a**) Distribution of TAM concentrations in whole venous blood and in plasma. The means were not significantly different (*P* = 0.325); (**b**) Distribution of ratios of plasma to venous blood TAM concentrations. In both graphs, the longer horizontal lines represent means (of concentrations or ratios), and the shorter horizontal lines represent standards of the mean (SEM).

Given that plasma is considered the gold standard for use when measuring TAM concentrations, we compared TAM concentrations in the plasma and in the venous blood of all participants. The mean concentrations of TAM in plasma and venous blood were 89.31 ng/mL and 98.31 ng/mL, respectively (Fig. 1a). The difference in these means was -9.00 (95% confidence interval [CI] − 27.38 to 9.38) ng/mL, and this difference was not statistically significant (*P* = 0.325). The ratio of plasma to venous blood TAM concentrations was also determined for each participant; the mean plasma-to-blood TAM ratio for the group was 0.914 (Standard Deviation [SD], 0.108; Standard Error of Mean [SEM], 0.027) (Fig. 1b).

In addition, we observed a substantial variation in the TAM concentrations among the participants, despite all participants taking the same 20 mg dose of TAM (Fig. 1a). In plasma, TAM ranged from 45.0 to 135.0 (SD, 24.71; SEM, 6.18) ng/mL in the group. In venous blood, TAM ranged from 49.0 to 140.0 (SD, 26.19; SEM, 6.55) ng/mL in the group.

**Figure 2:**
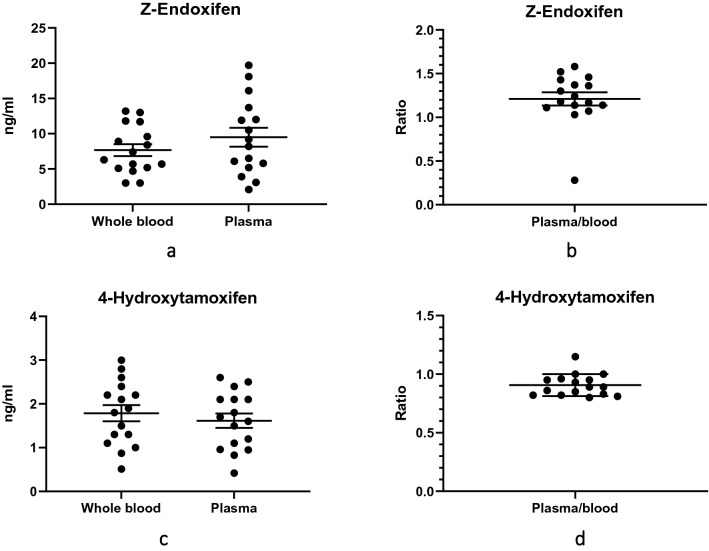
Z-endoxifen (END) and 4-Hydroxytamoxifen (4HT) concentrations in 16 participants with breast cancer taking tamoxifen 20 mg daily: (**a**) Distribution of END concentrations in venous blood and in plasma. The means were not significantly different (*P* = 0.257); (**b**) Distribution of ratios of plasma to venous blood END concentrations; (**c**) Distribution of 4HT concentrations in venous blood and in plasma. The means were not significantly different (*P* = 0.496); (**d**) Distribution of ratios of plasma to venous blood 4HT concentrations. The longer horizontal lines represent means (of concentrations or ratios), and the shorter horizontal lines represent standards of the mean (SEM).

We also compared END and 4HT concentrations in the venous blood and plasma of all participants. The mean concentrations of END in plasma and venous blood were 9.51 ng/mL and 7.67 ng/mL, respectively (Fig. 2a,b). The difference in these means was 1.84 (95% CI − 1.41 to 5.09) ng/mL, and this difference was not statistically significant (*P* = 0.257).

The mean concentrations of 4HT in plasma and venous blood were 1.62 ng/mL and 1.78 ng/mL, respectively (Fig. 2c,d). The difference between the mean venous blood and plasma concentrations of 4HT was − 0.17 (95% CI − 0.67 to 0.33) ng/mL, and this difference was not statistically significant (*P* = 0.496).

As with TAM, we also observed a substantial variation in the END and 4HT concentrations in the venous blood of the participants: 3.0 ng/mL to 13.2 ng/mL for END and 0.5 ng/mL to 3.0 ng/mL for 4HT (Fig. 2a,c).

### Capillary vs. venous blood concentrations of TAM, END, and 4HT

**Figure 3:**
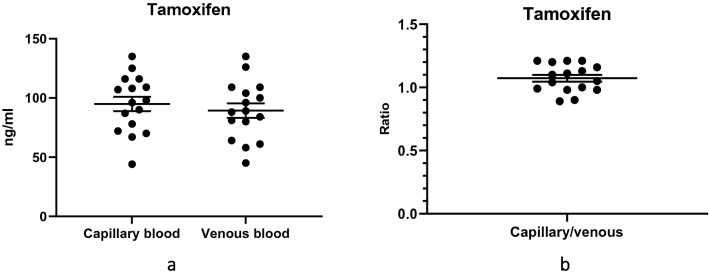
Tamoxifen (TAM) concentrations in 16 participants with breast cancer taking tamoxifen 20 mg daily: (**a**) Distribution of TAM concentrations in capillary blood and in venous blood. The means were not significantly different (*P* = 0.525); (**b**) Distribution of ratios of capillary blood to venous blood tamoxifen (TAM) concentrations. In both graphs, the longer horizontal lines represent means (of concentrations or ratios), and the shorter horizontal lines represent standards of the mean (SEM).

To determine the feasibility of using capillary blood to measure TAM concentrations, we compared the concentrations of TAM in the capillary blood and in the venous blood, and we also determined the ratio of capillary blood to venous blood TAM concentrations for each participant (Fig. 3). The difference between the mean TAM concentrations in capillary and venous blood for the group was − 5.56 (95% CI − 23.20 to 12.10) ng/mL, and this was not statistically significant (*P* = 0.525). The mean ratio of capillary to venous blood TAM concentrations for the group was 1.100 (SD, 0.110; SEM, 0.027), which represented a coefficient of variation of 10%.

The mean concentrations of END in capillary and venous blood for the group were 41.77 ng/mL and 7.67 ng/mL, respectively. Thus, the capillary concentration was 545% of the venous concentration of END. The difference between these means was 34.10 (95% CI 8.52 to 59.68) ng/mL, and the difference was statistically significant (*P* = 0.011). The mean concentrations of 4HT in capillary and venous blood were 0.99 ng/mL and 1.78 ng/mL, respectively. Thus, the capillary concentration was 56% of the venous concentration of 4HT. The difference between these means was − 0.79 (95% CI − 1.22 to − 0.36) ng/mL, and it was statistically significant (*P* < 0.001).

### Stability of analytes

The analysis of the feasibility of using the rhelise kit for capillary sampling and sample processing for measurements of TAM concentrations included determining whether the analytes in blood samples could be preserved in the extraction solution, be stored and/or shipped at various temperatures, and yet remain stable. Using the technique described in the Methods section of this manuscript, blood samples from a patient with breast cancer taking tamoxifen 20 mg were obtained, processed, and stored. It was determined that the initial TAM, END, and 4HT concentrations in this patient were 135 ng/mL, 5.7 ng/mL, and 3.0 ng/mL, respectively. When analyzed over time, the concentrations of TAM, END, and 4HT remained relatively stable, whether stored in the dark at refrigerator or room temperature, and when measured at both 7 days and 14 days after sampling (Table 2).

**Table 2:** Concentrations of tamoxifen (TAM), Z-endoxifen (END), and 4-hydroxytamoxifen (4HT) in a single patient with breast cancer receiving tamoxifen 20 mg daily, from whole blood samples stored at refrigerator and room temperatures in the dark, and analyzed 7 and 14 days after samples obtained, with results expressed as percentages of initial analyte concentrations.

Analyte	Storage temperatures, °C (± SD)	Time of analysis, days		
		0	7	14
Tamoxifen (TAM)	8 ± 2	100%	101%	105%
	20 ± 5	100%	102%	117%
Z-endoxifen (END)	8 ± 2	100%	102%	112%
	20 ± 5	100%	115%	115%
4-hydroxytamoxifen (4HT)	8 ± 2	100%	112%	106%
	20 ± 5	100%	110%	102%

SD, standard deviation.

## Discussion

The overarching goal of this project was to explore the feasibility of using an innovative capillary blood sampling kit to perform TDM of TAM and its metabolites. This was based on the concept that use of this kit for TDM might help empower patients with BC on TAM therapy, by enhancing their engagement, allowing modification and personalization of their treatment, and improving their overall sense of well-being. The results of this study suggest that venous blood can be used as an alternative to plasma to determine TAM, END, and 4HT concentrations, that capillary blood can be used as an alternative to whole venous blood to determine TAM concentrations, and that it is technically feasible to use an innovative kit containing a premixed extraction solution to assist with obtaining, processing, and storing capillary blood for TAM testing. However, we were unable to demonstrate that the kit and capillary blood can be used to reliably determine END and 4HT concentrations.

Recent technological advances have stimulated efforts to bring personalized medicine into clinical medical practice^11^. Personalized medicine involves the tailoring of treatment to the individual characteristics of each patient. The role that TDM might play in personalized medicine may be underappreciated. In fact, TDM can provide individualized information about patient drug levels relative to the therapeutic window of that agent, thus potentially reducing adverse effects, improving treatment outcomes, and reducing healthcare costs.

Historically, serum or plasma have been preferred over whole blood for TDM, because of ease of use in chemical analysis, simplicity of storage, and homogeneity compared with clotted blood. Attempts to use whole blood with early separation techniques and mass spectrometry instrumentation resulted in impaired resolution and inaccurate results. Consequently, plasma became the gold standard to use for testing. However, the contemporary techniques and instrumentation used routinely now have made it possible for laboratories to use blood instead of plasma. Yet, while whole venous blood or plasma sampling are still the most common approaches used for TDM measurements, capillary blood sampling may be an attractive alternative given that it is low-cost, easy-to-use, and can be done anywhere. Another option for TDM in this setting is dried blood sampling (DBS). DBS involves collecting blood from a fingertip and placing it on paper media, and use of this technique for determining TAM and END concentrations has been described^12^. Like capillary blood sampling, it is done without the need for venipuncture, can be performed anywhere, and requires only a small volume of blood. However, errors can be introduced if blood is blotted or smeared rather than drawn onto the filter paper by capillary action, and the process of extracting the analytes from the blood spot into a solution may lead to variations in results.

In our small preliminary study, we first set out to confirm that venous blood is feasible to use for TDM during TAM therapy, which we did by observing that the mean concentrations of TAM, END, and 4HT measured in venous blood did not differ significantly from the mean concentrations of these analytes measured in plasma. However, as noted above, there are barriers that make the use of whole blood sampling less than optimal for this type of patient testing, including that sampling is restricted to only the hours that laboratories are open, the logistics of scheduling patients for testing can be complicated, material and labor costs of obtaining and storing samples can be high, and many drugs levels are not tested frequently enough to allow routines or economies of scale to be developed in individual labs.

After confirming that venous blood was feasible to use for TDM in patients on TAM therapy, the next step in our study was to determine whether the concentrations of TAM and its metabolites in capillary blood would be similar to those in venous blood. We were able to confirm that the mean concentration of TAM measured in capillary blood did not differ significantly from that measured in venous blood. Conversely, we were unable to demonstrate that END and 4HT concentrations in capillary blood and venous blood samples did not differ. In fact, we identified large variations, including a mean capillary blood END concentrations that was over five times higher than that in venous blood, and a mean capillary blood 4HT concentration that was just over half of that in venous blood.

A post-hoc analysis initially suggested that the high END concentrations could have been caused by the absorption of END by the O-ring used to seal the capillary sampling vessels. However, after additional consideration, we determined that this was not the case. Instead, we suspect that a more likely cause of the high levels of capillary blood END was that END was more susceptible than TAM, within the respective internal standard solutions, to photodegradation^13^. Since the solutions used in the kit were produced in September 2019 and they were actually used in January 2020 for the study, it is possible that END photodegradation and other destructive reactions may have occurred after production of the END internal standard and before use of the kits. If the degradation process had occurred after use of the kit, degradation of the END in the internal standard and in the patient samples would have occurred at the same rate.

Based on the similarity of chemical structures of END and 4HT, we would have expected that the two should have behaved similarly concerning various kinds of degradation. However, unlike the END concentrations, the 4HT concentrations in the capillary blood samples were lower than in venous blood. Given that these are each complex molecules which are capable of reacting in different ways, potentially resulting in different degradation products and rearrangements, we cannot exclude that the peaks interpreted as 4HT and END during LC–MS/MS also had contributions from products that had similar chromatographic retention times to 4HT and END^14,15^. Thus, differences in the products of 4HT and END may have also contributed to the differences in the 4HT and END concentrations.

To explore the issues above in more detail, and make another attempt to validate the feasibility of serial assessments of TAM, END, and 4HT concentrations by capillary blood sampling, we are planning a prospective study that will also include patient-reported symptom scores and take into account other variables, such as age, comorbidities, and menopausal status (ClinicalTrials.gov Identifier: NCT05133674). We plan to attempt to mitigate the inconsistency of END and 4HT capillary blood results, including by using light-protected sampling vessels for the study. We anticipate that this next study will provide preliminary data to allow us to develop a future multicenter, randomized clinical trial of personalized dose monitoring and adjustment of adjuvant TAM therapy, with the goal of improving patient quality-of-life and breast cancer outcomes.

Despite the heterogeneity of some of the capillary blood results, we were able to demonstrate in all participants that it was feasible to use the items contained in the rhelise kit for the process of obtaining and preserving capillary blood samples for measurements of TAM. We were also able to show that the chosen extraction solution allowed analytes to be preserved and to remain relatively stable for up to 14 days after mixing and under temperatures ranging from 6 to 25 °C. These findings are important, because a potential benefit of capillary blood sampling is that it could facilitate the process of TDM, by enabling patients to obtain their own samples, and allowing them to do so at any time and place, including at the point-of-care or at home. The application of capillary sampling techniques to TDM of TAM could open up the possibility of home-based self-testing for patients with BC, many of whom may feel that they have already spent enough time in medical settings. This approach could be more convenient and cost-effective, allowing patients to avoid making frequent trips to hospital-based or independent laboratories, and it could also be a huge benefit for patients in rural and other sparsely populated regions that are far away from medical facilities. It might also reduce laboratory costs, given that samples could be stored and then run in weekly batches. The use of a capillary blood sampling technique for TDM of a commonly used medication like TAM could lead to the recasting of healthcare processes for patients with BC, resulting in more efficient and accessible care for these patients and in economic benefits for healthcare systems.

Another noteworthy finding of our study was the considerable variation in TAM, END, and 4HT concentrations in the blood of the women in our study. Variations in TAM and END concentrations have been linked by others to noncompliance with TAM therapy^16,17^. However, in our study all 16 participants explicitly confirmed that they had been compliant with the same standard tamoxifen 20 mg daily adjuvant therapy for a least a month, and the participant group had been on that regimen for a mean duration of 18.5 months. Also, unlike in some of these other studies, the analyte concentrations in our study varied widely. Specifically, TAM, END, and 4HT concentrations ranged from 49.0 ng/mL to 140.0 ng/mL, 3.0 ng/mL to 13.2 ng/mL, and 0.5 ng/mL to 3.0 ng/mL, respectively, in the venous blood of patients in our study group. This substantial variation in the TAM and metabolite concentrations suggests that patients taking the same standard dose of TAM may be experiencing a wide range of actual bioactivity from the medication. If so, this emphasizes the potential value of TDM during TAM therapy, potentially allowing personalized dose adjustments, and an opportunity to improve the efficacy and reduce the adverse effects experienced by patients. It also suggests that additional research is needed that involves the monitoring of TAM, END, and 4HT levels over time and that results in the development of therapeutic concentration ranges for each these substances.

To date, there is no evidence that TAM concentrations by themselves have predictive value in terms of efficacy or tolerability during TAM therapy. This fact, together with the potential bioactivity of TAM metabolites, was the rationale for including measurements of END and 4HT concentrations in our study. The metabolism of TAM involves a series of CYP450 enzymes that convert TAM to its two most active metabolites, END and 4HT, and this process may vary between patients^7^. Because these two metabolites have a 30 to 100 times greater binding affinity for the estrogen receptor than does TAM, and END achieves a 5 to 10 times higher plasma concentration than 4HT, END is thought to be the most active TAM metabolite^18^.

It has also been suggested that END concentrations may be the most important determinant of both the efficacy and toxicity of TAM therapy. In a retrospective study, patients on TAM therapy with END concentrations below 6 ng/mL experienced mild or no side effects relative to patients with END concentrations above 6 ng/mL^19^. Conversely, in a different retrospective study, patients with high END concentrations experienced more severe side effects as well as lower treatment compliance rates^20^. Yet despite these findings, direct correlations of TAM metabolite concentrations with TAM therapy toxicity have yet to be validated in prospective studies^18,21,22^. Thus, future studies are needed to measure TAM and metabolite concentrations as well as TAM therapy side effects, in order to determine the levels of correlation between them.

Direct correlations of concentrations of TAM or its metabolites with BC outcomes have also not been validated in prospective studies^23–25^. However, other evidence suggests that TAM metabolite concentrations may have some relationship with BC outcomes and prognosis. Based on retrospective studies, women with END concentrations below 6 ng/mL had a higher risk of BC relapse and lower survival rates than patients with END concentrations above 6 ng/mL^26,27^. Also, in a study involving a large cohort of ER-positive patients with BC taking TAM, the concentration of TAM did not impact the risk of BC recurrence, but the concentration of END, when in the bottom quintile (below 5.97 ng/mL), was significantly associated with an increased risk of BC recurrence^28^. In addition to END levels, 4HT levels may also play a role in BC outcomes, based on another retrospective study showing that low 4HT concentrations were associated with inferior BC-specific survival in a subgroup of premenopausal women on TAM therapy^29^. Interestingly, the results of the latter two studies suggest that END and 4HT concentrations may have a threshold rather than dose–response effect on BC outcomes in women on TAM therapy. This all suggests that the development and validation of an accurate capillary blood sampling and analysis method for END and 4HT remain important goals.

Patients with impaired cytochrome P2D6 (CYP2D6) metabolizer phenotype (i.e., lacking the critical enzymes for conversion of TAM to 4HT and of 4HT to END), and patients on drugs that compete with CYP2D6 (e.g., specific serotonin reuptake inhibitors), were noted to have END concentrations in the bottom quintile^28^. Because of results like these, there has been hope that phenotyping could be used as a tool to predict which patients might have abnormal TAM metabolism and therefore might be more likely to benefit from TDM of TAM and its metabolites^30,31^. However, according to others, the value of the use of phenotyping in this manner seems to apply to only the 20% of patients who carry two inactive CYP2D6 alleles; consequently, phenotyping seems to lack predictive value for the remaining 80% of patients^32^.

The heterogeneity of results of various studies with regards to the impact of TAM, END, and 4HT levels on TAM therapy efficacy and toxicity are somewhat surprising in light of the documented benefits of the therapy in reducing the risk of BC recurrence and death^2,3^. However, as a recently published in-depth review has suggested, TAM metabolite levels, along with other clinical and genetic variables, could potentially be used in the future to help develop personalized TAM dosing^11^. Thus, there remains a strong possibility that as more information is gathered, TDM involving TAM and its metabolites is likely to play some role in personalizing TAM therapy dosing and improving the efficacy and tolerability of TAM therapy. If so, the ability to obtain reliable TAM, END, and 4HT levels easily, through the use of capillary blood sampling, will be important.

### Limitations

The primary limitation of this study was the small size of our population. However, this was purposeful in that this was intended only as a preliminary feasibility study. The findings from this study will allow us and others to move forward with additional efforts to employ capillary blood sampling in the process of determining the concentrations of TAM and its metabolites in larger populations, and in determining the levels of correlation between these substances and TAM toxicity and efficacy. Also, the inconsistent results that we observed in the capillary blood concentrations of 4HT and END have encouraged us to pursue exploration of other technical and processing variables in a future study.

## Conclusions

Although TAM has been on the market for several decades and is the most widely used medication for patients with BC, TAM levels are not routinely measured and TAM doses are not usually adjusted in patients. Our experience suggests that it may be feasible and reliable to do TDM during TAM therapy using an innovative kit and capillary blood testing. There is additional work to be done before this approach can be applied reliably to measuring END and 4HT concentrations. The preliminary data from this study has aided us in developing a multicenter, prospective, randomized clinical trial of personalized TAM, END, and 4HT level monitoring and TAM dose adjustments over time, with the goal of enhancing the quality-of-life and outcomes of patients with BC.

## Methods

### Participants and sampling

This study was performed in accordance with the Declaration of Helsinki and was approved by the Regional Ethics Review Board of Karolinska Institute (Dnr 2019-02745).

#### Participant selection and study design

Patients who were participants in the single-center Tailor Dose II study (EudraCT No. 2017-000641-44) at the Karolinska University Hospital Breast Centre were eligible for this small pilot study. From January 17, 2020 to January 30, 2020, eligible patients were informed of the pilot study and written informed consent was obtained from those electing to participate. Patients were included who had BC and had been receiving an ongoing course of adjuvant TAM 20 mg daily for at least a month. Sampling occurred once for each participant, between Monday and Friday, from 0900 to 1500 h, at the Breast Centre. For each participant, time and date of sampling, age, and duration of TAM treatment were noted. In addition, participant functioning, and TAM side effects were identified using a variety of questionnaires.

#### Participant sampling techniques

Whole venous blood, plasma, and capillary blood samples were taken at the same session for each patient to allow direct comparisons of the results from each sampling technique. First, two venous samples were collected in EDTA tubes. One tube was centrifuged, and the isolated plasma was set aside and frozen (− 20 °C), while the other tube of whole blood was frozen (− 20 °C) without processing. Next, two capillary samples (50 µL each) were obtained. For this process, the finger was disinfected with a 70% isopropanol cloth, air-dried, and warmed. The fingertip was then pricked using a single-use automatic 1.8 mm safety lancet (Sarstedt AG & Co KG, Germany) and capillary blood was collected using a capillary Minivette POCT 50 µL (Sarstedt AG & Co KG, Germany). Capillary blood samples were immediately transferred into sampling vessels, in which they were mixed with 150 µL of the extraction solution. The vessels were vigorously shaken by hand, sealed with an O-ring, and then stored in the dark at room temperature (22 °C). All items used to obtain the capillary blood samples were from a rhelise kit. This kit is a patent-pending sampling kit intended to be used by laymen in a home environment. The kit consists of a lancet, a capillary, and a vial with an extraction solution. The extraction solution is optimized to keep analytes stable for more than 14 days at room temperature without the need for cold storage or additional extraction procedures at the laboratory.

### Materials and methods

#### Chemicals

##### Reference standards

For the preparation of stock solutions to be used in the study, TAM (tamoxifen > 99%, product #T5648, batch #BCBW6527) and 4HT (4-hydroxytamoxifen ≥ 98% Z-isomer, product #H7904, batch #067M4003V) were both obtained from Sigma-Aldrich (Sweden). END (Z-endoxifen 99.3% E/Z Mixture 50/50, product #D21865, batch #HY-18719A/CS-5098) was obtained from Med Chem Express Europe (Sweden).

##### Internal reference standards

^13^C_6_ TAM (^13^C_6_ tamoxifen 99%, product #T006002, batch #7-JMR-144-1) was obtained from Toronto Research Chemicals (Canada). ^13^C_6_ END (^13^C_6_ Z-endoxifen 99% E/Z Mixture 50/50, batch #NC029-39-2) and ^13^C_6_ 4HT (^13^C_6_ 4-hydroxytamoxifen ≥ 98%, batch #NC029-39-2) were custom-synthesized by Novandi Chemicals (Sweden) and obtained from Redhot Diagnostics (Sweden).

#### Preparation of solutions for LC–MS/MS method

##### Stock standard solutions (Solution I)

Standard solutions of 1.00 mg/mL TAM, END, and 4HT were prepared by dissolving approximately 5 mg of each solid chemicals in an exact volume of acetonitrile (ACN) fortified with 0.2% formic acid to yield the desired concentration. Aliquots from each stock solution were further diluted in aqueous ACN (1/4 v/v) to yield 1.00 µg/mL solutions of TAM, END, and 4HT.

##### Stock internal standard solutions (Solution II)

Internal standard solutions of 1.00 mg/mL ^13^C_6_ TAM, ^13^C_6_ END and ^13^C_6_ 4HT were prepared from the solid components dissolved in ACN with 0.2% formic acid to yield the desired concentrations, as described in the previous step.

##### Protein extraction solutions (Solution III)

Extraction solutions were prepared by diluting 20 µL aliquots of the stock internal standard solutions (consisting of ^13^C_6_ TAM, ^13^C_6_ END, and ^13^C_6_ 4HT) into ACN fortified with 200 mL of 0.2% formic acid, to yield 10.0 ng/mL extraction solutions of each isotope-substituted standard.

##### Quality control (QC) solutions (Solution IV)

QC solutions were prepared by diluting 10 µL of each stock standard of TAM, END, and 4HT, respectively, in 970 µL frozen-thawed human whole blank blood (commercially available, from healthy donors), to yield 10.0 µg/mL solutions. Each 10.0 µg/mL solution was further sequentially diluted with blank blood to yield control blood samples of 100 ng/mL, 10.0 ng/mL, and 1.00 ng/mL for each of the 3 analytes.

##### Calibration solutions (Solution V)

Calibration solutions were prepared by first diluting 10 µL of each stock standard of TAM, END, and 4HT, respectively, in 970 µL frozen-thawed human whole blank blood to yield calibration stock solutions containing 10.0 µg/mL of each analyte. These calibration stock solutions were further sequentially diluted with lysed whole blank blood to yield calibration solutions of 200, 100, 20.0, 10.0, 2.00, 1.00, 0.200 and 0.100 ng/mL for each analyte.

#### High-performance LC–MS/MS method

A Waters Premier Triple Quadrupole Mass Spectrometer (Waters Ltd, United Kingdom) was used for all analyses. Separation was performed using a Rheos Alliance UPLC Binary Pump (Flux Instruments AG, Switzerland) with an XSelect HSS T3 column (50 mm × 2.1 mm, 2.5 µm) controlled by MassLynx software (both Waters Ltd, United Kingdom). The mobile phases of processing involved 0.1% formic acid in water (eluent A) and 0.1% formic acid in ACN (eluent B), running at a flow rate of 0.40 mL/min. The initial composition of 95% eluent A was maintained for 0.5 min and then the composition was decreased to 40% eluent A for the next 6.5 min and to 5% eluent A for another 2 min, resulting in a time of analysis of 9 min. Mass spectrometer conditions were optimized with a capillary voltage of + 3000 V, desolvation temperature 300 °C, desolvation flow 600 L/h and ESI+, 30 eV (for END, ^13^C_6_ END) and 27 eV (for all others), with *m/z* values of 372 → 72.3 for TAM, 374 → 58.2 for END, 388 → 72.3 for 4HT, 378 → 72.3 for ^13^C_6_ TAM, 380 → 58.2 for ^13^C_6_ END, and 394 → 72.3 for ^13^C_6_ 4HT.

#### Analysis of stock standard solutions (Solutions I and II above)

Stock solutions for the calibration curves were prepared in duplicates by separate weigh-ins of TAM, END, 4HT, ^13^C_6_ TAM, ^13^C_6_ END, and ^13^C_6_ 4HT, mixed with human blank blood to final concentrations of 200, 100, 20.0, 10.0, 2.00, 1.00, 0.200 and 0.100 ng/mL, which were then analyzed with the LC–MS/MS method described above to obtain the calibration curves.

#### Sample preparation—protein extraction

Protein extraction was performed in tubes by adding 50 µL of blood samples from participants to 150 µL of the protein extraction solutions (Solutions III above) containing 1.00 ng/mL of the standards ^13^C_6_ TAM, ^13^C_6_ END, and ^13^C_6_ 4HT, respectively. The tubes were then vortexed for 10 s and centrifuged in an Eppendorf centrifuge for 10 min at 10 °C. After centrifugation, 150 µL of each supernatant was transferred to a conical autosampler glass vial, evaporated to dryness under a gentle stream of nitrogen, and reconstituted in 60 µL of 20% ACN in water.

#### LC–MS/MS method validation

Method validation was done in accordance with the document *Bioanalytical Method Validation Guidance for Industry*^33^. Selectivity, carry-over, calibration curves, lower limit of quantification (LLOQ), accuracy, precision, matrix effects, and stability were evaluated.

##### Preparation of batches

Triplicate validation batches of TAM, END, and 4HT were prepared, and these batches were then analyzed with the described LC–MS/MS method on three separate occasions. Each validation batch consisted of QC solutions (Solutions IV above), calibration solutions (Solutions V above), and blank solvent solutions to measure carry-over effects between different runs.

##### Selectivity and carry-over

**Figure 4:**
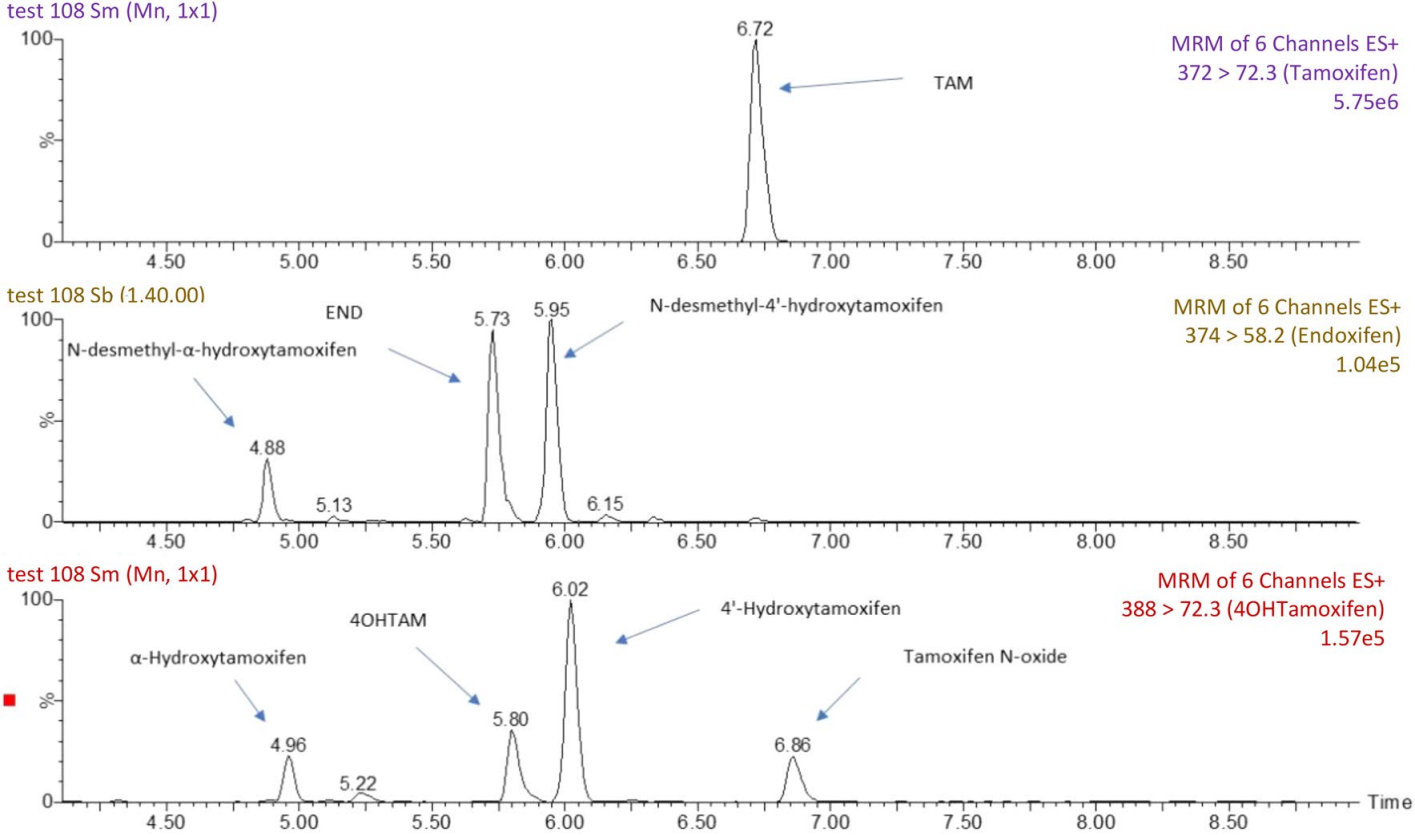
Mass spectrometry results demonstrating fragmentation pathways for tamoxifen (TAM), Z-endoxifen (END), and 4-hydroxytamoxifen (4HT), respectively. The graphs show the separate peaks for each analyte and demonstrate that there is no overlap between the peaks of these analytes or of any other TAM metabolites.

As part of the validation process, experiments were performed using patient blood samples to determine whether TAM, END, and/or 4HT co-eluted or interfered with any other metabolites of TAM. The mass spectrometric peaks for TAM, END, and 4HT were well separated without overlap (Fig. 4). The selectivity and carry-over were determined to be adequate for the intended analyses.

##### Calibration curves

**Figure 5:**
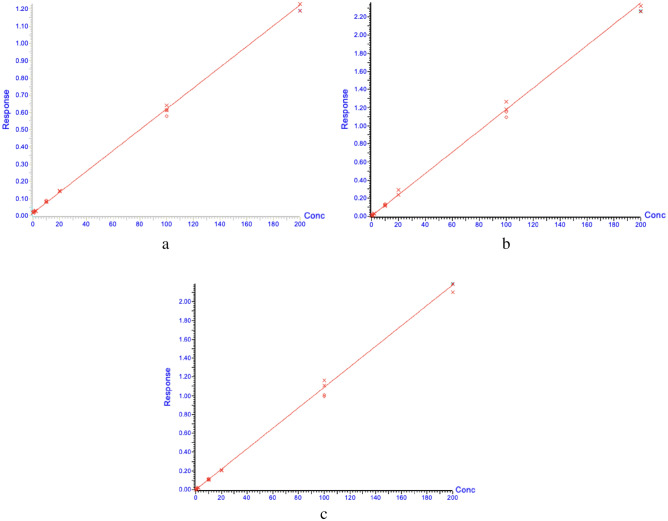
Concentration–response calibration curves for (**a**) tamoxifen (TAM), (**b**) Z-endoxifen (END), and (**c**) 4-hydroxytamoxifen (4HT). Calibration curve linearity was r = 0.999 and r^2^ = 0.999 for TAM, r = 0.999 and r^2^ = 0.997 for END, and r = 0.999 and r^2^ = 0.999 for 4HT.

Concentration–response calibration curves for TAM, END, and 4HT were constructed based on two QC samples (Solutions IV above) and two analyses of each calibration solution (Solutions V above) at each concentration (Fig. 5). The prepared calibration solutions were analyzed and a regression line was fitted to the data for each analyte. Linearity of the calibration curves was identified from 1 to 200 ng/mL for TAM and from 0.2 to 200 ng/mL for both END and 4HT.

##### Lower limit of quantification (LLOQ)

Based on the linearity results of the calibration curves, the LLOQ for the method was determined to be 1.0 ng/mL for TAM and 0.20 ng/mL for both END and 4HT.

#### Precision and accuracy

Using the QC solutions (Solutions IV) and the calibration standard solutions (Solutions V above) for TAM, END, and 4HT, the precision, accuracy, and deviations from the nominal concentrations of the calibration standard solutions were determined. The concentration range for TAM was 1 ng/mL to 100 ng/mL, and the concentration ranges for both END and 4HT were 0.20 ng/mL to 100 ng/mL. The coefficients of variation were all less than 10%. The levels of precision and accuracy fulfilled FDA requirements for quantitative bioanalysis^33^.

#### Stability testing

To determine the stability of each particular analyte (TAM, END, or 4HT) in a whole blood sample, a 50 µL sample aliquot was transferred to an Eppendorf 0.5 mL tube containing 150 µL of extraction solution (without internal standards for TAM, END, and 4HT). Each tube was then vortexed for 10 s and placed in an Eppendorf Thermomixer, where it was agitated for 10 min at 10 °C. Each tube was stored in the dark at either room temperature (20 °C) or in a refrigerator (8 °C), for either 7 or 14 days. After retrieval from storage, 150 µL of protein precipitation solution (Solutions III above) containing the internal standards of either TAM, END, or 4HT was added to the respective tube and the solution was briefly mixed. The solutions were then centrifuged at 20,100×*g* for 10 min at 10 °C. After centrifugation, 300 µL of the supernatant was collected and transferred to a conical autosampler glass vial. The protein precipitation solution was evaporated to dryness and reconstituted in 60 µL of 20% ACN in water, after which each was analyzed. The stability period that was selected for analysis was an estimate, based on what was deemed to be adequate time to accomplish the logistics of getting samples from patients, shipping them, and then analyzing them at the laboratory. It was estimated that a period of 7 days between sampling and analysis, including shipping, should be sufficient, but in case of unforeseen events, samples should ideally remain stable for up to 14 days.

#### Statistical methods

Demographic and clinical data, as well as ratios, are reported using either medians or means and either ranges or interquartile ranges (IQR), which were obtained using Excel. Plasma, venous whole blood, and capillary blood concentrations of TAM, END, and 4HT were presented as ratios for individuals, group means, group mean ratios, standard deviations (SD), and standards of the mean (SEM). Comparisons of the means were performed using the two-sided t-test and *P* values reported. Coefficient of variation (CV) was calculated by dividing SD by the mean and multiplying by 100. To evaluate correlations between analyte concentrations in plasma, whole blood, and capillary blood, a non-parametric Passing-Bablok regression analysis was performed for each analyte separately, using MedCalc (MedCalc Software bvba, Belgium). Statistical significance was defined at the 5% (*P* ≤ 0.05) level.

## Data Availability

The datasets generated during the current study are not publicly available due to the privacy policy concerning private information of active army personnel, but they are available from the corresponding author upon reasonable request.
